# Supplementary material for: Structure-Directing Interplay between Tetrel and Halogen Bonding in Co-Crystal of Lead(II) Diethyldithiocarbamate with Tetraiodoethylene
Source: Int J Mol Sci. 2022 Oct 6;23(19):11870. doi: 10.3390/ijms231911870 (PMC9569466; doi:10.3390/ijms231911870)
Supplement: Supplementary file 1 [file ijms-23-11870-s001.zip › ijms-1951238-supplementary.pdf]

Electronic supplementary information

Structure-directing Interplay between Tetrel and Halogen Bonding in Cocrystal of Lead(II) Diethyldithiocarbamate with Tetraiodoethylene

Lev E. Zelenkov,[1,2] Daniil M. Ivanov,[1] Ilya A. Tyumentsev,[3] Yulia A. Izotova,[1] Vadim Yu. Kukushkin,[1,4]* Nadezhda A. Bokach*[1]

[1]*Institute of Chemistry, Saint Petersburg State University, Universitetskaya Nab. 7/9, 199034 Saint Petersburg, Russian Federation*

[2] *School of Physics and Engineering, ITMO University, 191002 Saint Petersburg, Russian Federation*

[3]*A. E. Favorsky Institute of Chemistry, Siberian Branch of the Russian Academy of Sciences, 664033 Irkutsk, Russia*

[4]*Institute of Chemistry and Pharmaceutical Technologies, Altai State University, 656049 Barnaul, Russian Federation*

Crystallographic data

Table S1. Crystal data and structure refinement for **1**·½C_2I_4

CSD code	2205815
Empirical formula	$C_{11}H_{20}I_2N_2PbS_4$
Formula weight	769.52
Temperature, K	100(2)
Crystal system	monoclinic
Space group	$P2_1/n$
a, Å	14.3805(4)
b, Å	8.4641(3)
c, Å	17.0782(8)
α, °	90
β, °	90.839(4)
γ, °	90
Volume, $Å^3$	2078.50(14)
Z	4
ρ_{calc}, g/cm^3	2.459
μ, mm^{-1}	11.483
F(000)	1408.0
Crystal size, mm^3	0.21 × 0.17 × 0.14
Radiation	MoKα (λ = 0.71073)
2Θ range for data collection, °	5.372 to 54.998
Index ranges	$-18 \le h \le 17$, $-10 \le k \le 10$, $-22 \le l \le 19$
Reflections collected	10204
Independent reflections	4761 [R_{int} = 0.0266, R_{sigma} = 0.0378]
Data/restraints/parameters	4761/0/185
Goodness-of-fit on F^2	1.022
Final *R* indexes [$I \ge 2\sigma(I)$]	R_1 = 0.0226, wR_2 = 0.0405
Final *R* indexes [all data]	R_1 = 0.0286, wR_2 = 0.0424
Largest diff. peak/hole, e·$Å^{-3}$	0.82/–0.71

Table S2. Parameters of short contacts in the structure of **1**·½C_2I_4.

**Contact**	**Type**	**Distance, Å**	**Nc[1]**	**Angle, °**
S3–Pb1···S1	TeB	3.2526(10)	0.85	140.85(3)
S1–Pb1···S3	TeB	3.3860(10)	0.88	163.57(3)
C1S–I2S···S2	HaB	3.2500(11)	0.86	174.52(8)
C1S–I1S···S1	HaB	3.3181(9)	0.88	173.02(9)

[1] Nc is normalized contact, Nc = d/$\sum_{vdW}$; $\sum_{vdW}$ is Bondi van der Waals radii sum: $\sum_{vdW}$(Pb + S) = 3.82 Å; $\sum_{vdW}$(I+S) = 3.78 Å.

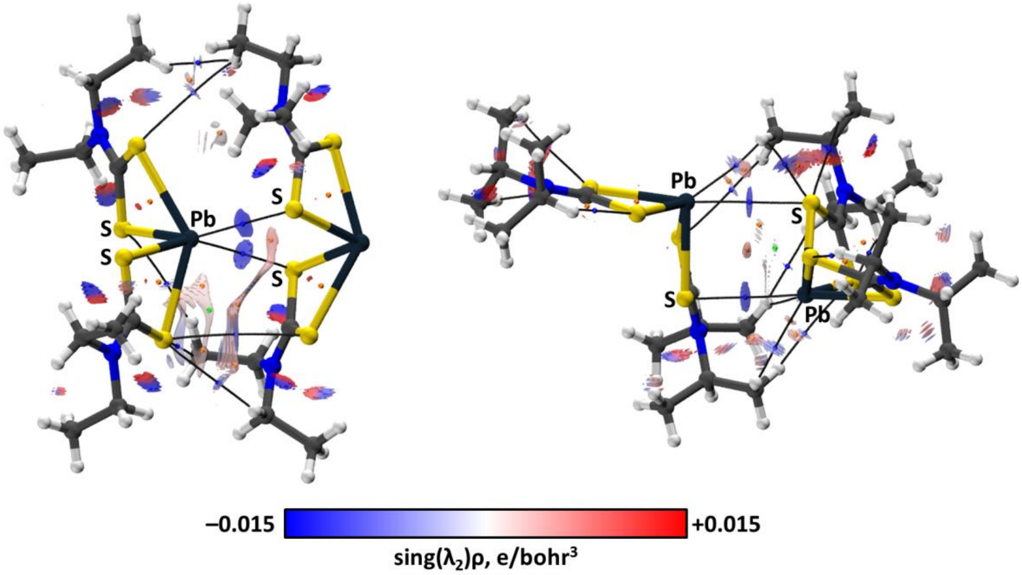

Figure S1. Visualization of QTAIM topological and NCI analyses for the model clusters (**1**)$_2$ (left) and ([Pb(S$_2$CN^{n}Pr$_2$)$_2$])$_2$ (right) from from PBETCA02 and IPTCPB01 structures, respectively, exhibiting two different Pb···S TeBs in both cases. Blue dots correspond to (3; –1) bond critical points, orange dots to (3; +1) ring critical points, bond paths are shown as black lines. For noncovalent interactions, RDG = 0.35 e$^{-1/3}$ half-transparent surfaces are colored from blue (sign(λ_2)ρ(**r**) = –0.015 e/bohr^3) to red (sign(λ_2)ρ(**r**) = +0.015 e/bohr^3) through white.

Table S3. Parameters in (3, –1) bond critical points (the electron density with sign of λ_2 sign(λ_2)ρ(**r**) in e/bohr^3, Laplacian of electron density $\nabla^2\rho$(**r**) in e/bohr^5, the local electronic energy density H$_b$, local electronic potential energy density V(**r**), local electronic kinetic energy density G(**r**) in hartrees/bohr^3) corresponding to the Pb···S TeBs in both crystal and cluster models as well as Wiberg bond indexes (WBI) calculated for the cluster model.

**Structure**	**Bond**	**Model**	**sign(λ_2)ρ(r)**	**$\nabla^2\rho$(r)**	**G(r)**	**V(r)**	**H$_b$**	**WBI**
PBETCA02	Pb1···S11	*crystal*	–0.014	0.029	0.007	–0.006	0.001	
		cluster	–0.015	0.028	0.007	–0.006	0.000	0.08
	Pb1···S12	*crystal*	–0.012	0.026	0.006	–0.005	0.001	
		cluster	–0.013	0.024	0.006	–0.005	0.000	0.07
IPTCPB01	Pb1···S2	*crystal*	–0.013	0.027	0.006	–0.006	0.001	
		cluster	–0.014	0.026	0.006	–0.006	0.000	0.07
	Pb1···S4	*crystal*	–0.013	0.026	0.006	–0.005	0.001	
		cluster	–0.013	0.024	0.006	–0.005	0.000	0.08

Table S4. Cartesian coordinates for cluster models.

	(**1**)·C_2I_4 (I1S···S1)		
Pb	6.808500	6.270459	7.862131
S	8.608540	4.346315	7.143045
S	8.409535	6.376768	10.107161
S	8.865805	8.067895	7.673437
S	6.940551	5.471279	4.952830
N	10.268836	8.266886	9.937081
C	9.288640	7.630386	9.310036
N	8.506747	3.326391	4.666972
C	11.034608	9.354523	9.298083
H	10.465815	9.815817	8.662842
H	11.306538	9.994409	9.976015
C	8.059065	4.277756	5.483222
C	10.699045	7.907162	11.296018
H	10.508360	6.969340	11.453121
H	11.657124	8.034124	11.371154
C	9.411944	2.242140	5.112665
H	9.387699	2.181199	6.080895
H	9.102313	1.397423	4.750646
C	10.000617	8.742569	12.344507
H	10.400783	8.580905	13.201741
H	10.085836	9.672773	12.122514
H	9.069748	8.504728	12.375245
C	12.267453	8.834828	8.580875
H	12.796382	8.313439	9.188794
H	11.997887	8.287200	7.839761
H	12.785564	9.575436	8.258132
C	8.036778	3.204508	3.273540
H	7.869489	4.089007	2.913229
H	8.729167	2.789767	2.735634
C	6.771207	2.379259	3.181328
H	6.057059	2.835474	3.633851
H	6.537366	2.253143	2.259204
H	6.914712	1.524384	3.594576
C	10.843654	2.485906	4.661849
H	11.164514	3.306924	5.042652
H	11.400192	1.759686	4.955562
H	10.873501	2.543462	3.703864
I	14.529448	2.855618	6.404834
I	16.734354	3.858021	8.981487
C	14.747439	3.905336	8.232518
I	13.981479	5.608482	10.671535
I	11.776574	4.606079	8.094882
C	13.763489	4.558764	8.843852
	(**1**)·C_2I_4 (I2S···S2)		
I	14.529448	2.855618	6.404834
I	16.734354	3.858021	8.981487
C	14.747439	3.905336	8.232518

I	13.981479	5.608482	10.671535
I	11.776574	4.606079	8.094882
C	13.763489	4.558764	8.843852
Pb	13.873713	6.425691	16.400316
S	15.673753	8.349835	15.681230
S	15.474749	6.319382	18.645346
S	15.931019	4.628255	16.211622
S	14.005765	7.224871	13.491015
N	17.334050	4.429264	18.475265
C	16.353854	5.065764	17.848221
N	15.571961	9.369759	13.205156
C	18.099822	3.341627	17.836267
H	17.531028	2.880333	17.201027
H	18.371752	2.701741	18.514199
C	15.124278	8.418394	14.021407
C	17.764259	4.788988	19.834203
H	17.573573	5.726810	19.991305
H	18.722338	4.662026	19.909339
C	16.477158	10.454010	13.650849
H	16.452912	10.514951	14.619080
H	16.167526	11.298727	13.288830
C	17.065830	3.953581	20.882692
H	17.465997	4.115245	21.739925
H	17.151050	3.023377	20.660699
H	16.134962	4.191422	20.913429
C	19.332667	3.861322	17.119060
H	19.861595	4.382711	17.726979
H	19.063101	4.408950	16.377946
H	19.850777	3.120714	16.796317
C	15.101992	9.491642	11.811724
H	14.934702	8.607143	11.451413
H	15.794381	9.906383	11.273819
C	13.836420	10.316891	11.719512
H	13.122273	9.860677	12.172036
H	13.602580	10.443007	10.797388
H	13.979926	11.171766	12.132760
C	17.908867	10.210244	13.200033
H	18.229728	9.389226	13.580836
H	18.465406	10.936464	13.493747
H	17.938715	10.152688	12.242049
	$(\mathbf{1})_2$ (Pb1···S1)		
Pb	7.321928	2.193641	9.214238
S	5.521888	4.117785	9.933324
S	5.720892	2.087332	6.969208
S	5.264623	0.396205	9.402932
S	7.189876	2.992821	12.123539
N	3.861592	0.197214	7.139288
C	4.841787	0.833714	7.766333
N	5.623680	5.137709	12.409397
C	3.095820	-0.890423	7.778286

H	3.664613	-1.351717	8.413527
H	2.823890	-1.530309	7.100354
C	6.071363	4.186344	11.593147
C	3.431383	0.556938	5.780351
H	3.622068	1.494760	5.623248
H	2.473304	0.429976	5.705215
C	4.718483	6.221960	11.963704
H	4.742729	6.282901	10.995474
H	5.028115	7.066677	12.325723
C	4.129811	-0.278469	4.731862
H	3.729644	-0.116805	3.874628
H	4.044591	-1.208673	4.953855
H	5.060679	-0.040628	4.701124
C	1.862974	-0.370728	8.495494
H	1.334046	0.150661	7.887575
H	2.132541	0.176900	9.236608
H	1.344864	-1.111336	8.818237
C	6.093649	5.259592	13.802829
H	6.260939	4.375093	14.163140
H	5.401260	5.674333	14.340735
C	7.359221	6.084841	13.895041
H	8.073368	5.628627	13.442518
H	7.593062	6.210957	14.817165
H	7.215716	6.939716	13.481793
C	3.286774	5.978194	12.414520
H	2.965913	5.157176	12.033717
H	2.730236	6.704414	12.120807
H	3.256926	5.920638	13.372505
Pb	6.808500	6.270459	7.862131
S	8.608540	4.346315	7.143045
S	8.409535	6.376768	10.107161
S	8.865805	8.067895	7.673437
S	6.940551	5.471279	4.952830
N	10.268836	8.266886	9.937081
C	9.288640	7.630386	9.310036
N	8.506747	3.326391	4.666972
C	11.034608	9.354523	9.298083
H	10.465815	9.815817	8.662842
H	11.306538	9.994409	9.976015
C	8.059065	4.277756	5.483222
C	10.699045	7.907162	11.296018
H	10.508360	6.969340	11.453121
H	11.657124	8.034124	11.371154
C	9.411944	2.242140	5.112665
H	9.387699	2.181199	6.080895
H	9.102313	1.397423	4.750646
C	10.000617	8.742569	12.344507
H	10.400783	8.580905	13.201741
H	10.085836	9.672773	12.122514
H	9.069748	8.504728	12.375245

C	12.267453	8.834828	8.580875
H	12.796382	8.313439	9.188794
H	11.997887	8.287200	7.839761
H	12.785564	9.575436	8.258132
C	8.036778	3.204508	3.273540
H	7.869489	4.089007	2.913229
H	8.729167	2.789767	2.735634
C	6.771207	2.379259	3.181328
H	6.057059	2.835474	3.633851
H	6.537366	2.253143	2.259204
H	6.914712	1.524384	3.594576
C	10.843654	2.485906	4.661849
H	11.164514	3.306924	5.042652
H	11.400192	1.759686	4.955562
H	10.873501	2.543462	3.703864
	($\mathbf{1}$)$_2$ (Pb1···S3)		
Pb	6.808500	6.270459	7.862131
S	8.608540	4.346315	7.143045
S	8.409535	6.376768	10.107161
S	8.865805	8.067895	7.673437
S	6.940551	5.471279	4.952830
N	10.268836	8.266886	9.937081
C	9.288640	7.630386	9.310036
N	8.506747	3.326391	4.666972
C	11.034608	9.354523	9.298083
H	10.465815	9.815817	8.662842
H	11.306538	9.994409	9.976015
C	8.059065	4.277756	5.483222
C	10.699045	7.907162	11.296018
H	10.508360	6.969340	11.453121
H	11.657124	8.034124	11.371154
C	9.411944	2.242140	5.112665
H	9.387699	2.181199	6.080895
H	9.102313	1.397423	4.750646
C	10.000617	8.742569	12.344507
H	10.400783	8.580905	13.201741
H	10.085836	9.672773	12.122514
H	9.069748	8.504728	12.375245
C	12.267453	8.834828	8.580875
H	12.796382	8.313439	9.188794
H	11.997887	8.287200	7.839761
H	12.785564	9.575436	8.258132
C	8.036778	3.204508	3.273540
H	7.869489	4.089007	2.913229
H	8.729167	2.789767	2.735634
C	6.771207	2.379259	3.181328
H	6.057059	2.835474	3.633851
H	6.537366	2.253143	2.259204
H	6.914712	1.524384	3.594576
C	10.843654	2.485906	4.661849

H	11.164514	3.306924	5.042652
H	11.400192	1.759686	4.955562
H	10.873501	2.543462	3.703864
Pb	7.321928	10.657741	9.214238
S	5.521888	12.581885	9.933324
S	5.720892	10.551432	6.969208
S	5.264623	8.860305	9.402932
S	7.189876	11.456921	12.123539
N	3.861592	8.661314	7.139288
C	4.841787	9.297814	7.766333
N	5.623680	13.601809	12.409397
C	3.095820	7.573677	7.778286
H	3.664613	7.112383	8.413527
H	2.823890	6.933791	7.100354
C	6.071363	12.650444	11.593147
C	3.431383	9.021038	5.780351
H	3.622068	9.958860	5.623248
H	2.473304	8.894076	5.705215
C	4.718483	14.686060	11.963704
H	4.742729	14.747001	10.995474
H	5.028115	15.530777	12.325723
C	4.129811	8.185631	4.731862
H	3.729644	8.347295	3.874628
H	4.044591	7.255427	4.953855
H	5.060679	8.423472	4.701124
C	1.862974	8.093372	8.495494
H	1.334046	8.614761	7.887575
H	2.132541	8.641000	9.236608
H	1.344864	7.352764	8.818237
C	6.093649	13.723692	13.802829
H	6.260939	12.839193	14.163140
H	5.401260	14.138433	14.340735
C	7.359221	14.548941	13.895041
H	8.073368	14.092727	13.442518
H	7.593062	14.675057	14.817165
H	7.215716	15.403816	13.481793
C	3.286774	14.442294	12.414520
H	2.965913	13.621276	12.033717
H	2.730236	15.168514	12.120807
H	3.256926	14.384738	13.372505
	($\mathbf{1}$)$_2$ (PBETCA02)		
Pb	5.099643	5.773751	6.180745
S	7.619179	6.485435	5.406823
S	5.441960	8.517531	5.534952
N	8.027212	9.104641	5.239749
C	7.108921	8.154561	5.381745
C	9.453586	8.813657	5.039626
H	9.852831	9.521121	4.473251
H	9.544433	7.953186	4.557967
C	10.220773	8.733023	6.349148

H	11.181017	8.671051	6.162343
H	9.933153	7.940775	6.848352
H	10.043389	9.536968	6.881393
C	7.687082	10.539694	5.240579
H	8.357115	11.033525	5.776495
H	6.802845	10.665144	5.667914
C	7.648699	11.109975	3.843867
H	6.930224	10.681236	3.335071
H	8.507293	10.945155	3.400348
H	7.485869	12.075680	3.889912
S	5.831083	3.237868	4.966884
S	4.076844	5.266224	3.671395
N	4.110248	2.681960	3.023443
C	4.619716	3.625028	3.815633
C	4.615292	1.293651	3.054168
H	5.594231	1.306483	3.201470
H	4.445193	0.865566	2.177577
C	3.950092	0.467444	4.156093
H	2.993347	0.378957	3.965161
H	4.071787	0.916003	5.018559
H	4.360557	-0.421985	4.189416
C	3.057540	2.955415	2.013691
H	2.549117	3.760891	2.282089
H	2.428527	2.192499	1.982726
C	3.650214	3.173945	0.629434
H	2.926591	3.230774	-0.029263
H	4.239324	2.424632	0.404291
H	4.164281	4.008250	0.622459
Pb	3.058705	5.912349	10.332685
S	5.578241	5.200665	9.558764
S	3.401022	3.168569	9.686893
N	5.986273	2.581459	9.391690
C	5.067983	3.531539	9.533686
C	7.412647	2.872443	9.191566
H	7.811893	2.164979	8.625192
H	7.503495	3.732914	8.709908
C	8.179835	2.953077	10.501088
H	9.140079	3.015049	10.314284
H	7.892215	3.745325	11.000293
H	8.002450	2.149132	11.033334
C	5.646144	1.146406	9.392520
H	6.316177	0.652575	9.928436
H	4.761907	1.020956	9.819854
C	5.607760	0.576125	7.995807
H	4.889286	1.004864	7.487012
H	6.466355	0.740945	7.552289
H	5.444931	-0.389580	8.041852
S	3.790144	8.448232	9.118824
S	2.035906	6.419876	7.823336
N	2.069309	9.004140	7.175384

C	2.578778	8.061072	7.967574
C	2.574354	10.392449	7.206108
H	3.553293	10.379617	7.353411
H	2.404255	10.820534	6.329517
C	1.909154	11.218656	8.308033
H	0.952408	11.307143	8.117102
H	2.030848	10.770097	9.170499
H	2.319619	12.108085	8.341357
C	1.016602	8.730685	6.165632
H	0.508179	7.925209	6.434030
H	0.387589	9.493601	6.134667
C	1.609276	8.512155	4.781375
H	0.885653	8.455326	4.122678
H	2.198386	9.261468	4.556232
H	2.123343	7.677850	4.774400
	($[Pb(S_2N^{n}Pr_2)_2])_2$ IPTCPB01		
Pb	4.019979	5.563543	16.015227
S	1.672947	5.536754	17.659359
S	6.855385	5.535830	16.330383
S	3.877470	3.580654	17.841348
S	5.057280	7.451956	17.647101
C	6.661457	7.001246	17.189221
C	2.284769	4.064535	18.302760
N	1.563642	3.336446	19.156612
N	7.713041	7.757888	17.527853
C	1.969715	2.012113	19.711304
H	1.223748	1.750102	20.324166
C	3.204948	2.059141	20.594241
H	4.006059	2.113726	20.033316
H	3.245692	1.247913	21.142700
H	3.160355	2.846014	21.178018
C	2.003228	0.925438	18.647625
H	1.239030	1.034609	18.045150
H	1.956568	0.046188	19.077667
H	2.836184	0.995139	18.136560
C	7.359163	8.955414	19.692606
H	6.424799	8.700961	19.842186
H	7.530324	9.828785	20.101873
H	7.948898	8.284430	20.095641
C	7.619973	9.032674	18.244590
H	8.600954	9.221624	18.300682
C	7.250887	10.197449	17.473838
H	7.682278	10.991041	17.851943
H	6.278105	10.311659	17.498768
H	7.540534	10.079879	16.545196
C	9.103516	7.347235	17.201686
H	8.893725	6.491499	16.730094
C	9.824268	6.791301	18.327690
H	10.058489	7.510153	18.953017
H	10.642913	6.356295	18.009833

H	9.260093	6.132913	18.784740
C	9.709621	8.039214	16.113077
H	9.119532	7.996385	15.329860
H	10.568176	7.620164	15.897017
H	9.854968	8.975569	16.360299
C	0.228235	3.802524	19.626126
H	0.100933	4.726282	19.264642
C	0.154811	3.907496	21.142700
H	0.244101	3.013970	21.537425
H	-0.707658	4.294635	21.402387
H	0.880879	4.480226	21.466790
C	-0.875917	2.940070	19.031962
H	-0.783143	2.917396	18.055538
H	-1.748666	3.318811	19.268797
H	-0.806739	2.029748	19.387214
Pb	4.113279	1.364643	15.147248
S	6.460311	1.337854	13.503116
S	1.277873	1.336930	14.832091
S	4.255788	-0.618246	13.321127
S	3.075979	3.253056	13.515373
C	1.471802	2.802346	13.973253
C	5.848490	-0.134365	12.859714
N	6.569616	-0.862454	12.005863
N	0.420217	3.558988	13.634621
C	6.163544	-2.186787	11.451171
H	6.909510	-2.448798	10.838309
C	4.928310	-2.139759	10.568234
H	4.127200	-2.085174	11.129158
H	4.887566	-2.950987	10.019774
H	4.972903	-1.352886	9.984457
C	6.130031	-3.273462	12.514850
H	6.894229	-3.164291	13.117324
H	6.176691	-4.152712	12.084808
H	5.297074	-3.203761	13.025914
C	0.774095	4.756514	11.469868
H	1.708459	4.502061	11.320288
H	0.602934	5.629885	11.060601
H	0.184361	4.085530	11.066833
C	0.513286	4.833774	12.917884
H	-0.467696	5.022724	12.861792
C	0.882371	5.998549	13.688636
H	0.450980	6.792141	13.310532
H	1.855153	6.112759	13.663706
H	0.592725	5.880979	14.617278
C	-0.970258	3.148335	13.960788
H	-0.760467	2.292599	14.432381
C	-1.691009	2.592401	12.834784
H	-1.925231	3.311253	12.209457
H	-2.509655	2.157395	13.152642
H	-1.126835	1.934013	12.377735

C	-1.576363	3.840314	15.049398
H	-0.986274	3.797485	15.832614
H	-2.434917	3.421264	15.265457
H	-1.721710	4.776669	14.802175
C	7.905023	-0.396376	11.536348
H	8.032326	0.527382	11.897833
C	7.978447	-0.291404	10.019774
H	7.889158	-1.184930	9.625050
H	8.840916	0.095735	9.760087
H	7.252380	0.281326	9.695685
C	9.009176	-1.258830	12.130512
H	8.916402	-1.281504	13.106937
H	9.881925	-0.880089	11.893678
H	8.939997	-2.169152	11.775260

Table S5. Cartesian coordinates for crystal models.

	**1**·½C_2I_4		
Pb	7.321928	2.193641	9.214238
S	5.521888	4.117785	9.933324
S	5.720892	2.087332	6.969208
S	5.264623	0.396205	9.402932
S	7.189876	2.992821	12.123539
N	3.861592	0.197214	7.139288
C	4.841787	0.833714	7.766333
N	5.623680	5.137709	12.409397
C	6.071363	4.186344	11.593147
C	3.431383	0.556938	5.780351
H	3.622068	1.494760	5.623248
H	2.473304	0.429976	5.705215
C	4.718483	6.221960	11.963704
H	4.742729	6.282901	10.995474
H	5.028115	7.066677	12.325723
H	1.334046	0.150661	7.887575
H	2.132541	0.176900	9.236608
C	6.093649	5.259592	13.802829
H	6.260939	4.375093	14.163140
H	5.401260	5.674333	14.340735
C	7.359221	6.084841	13.895041
H	8.073368	5.628627	13.442518
H	7.593062	6.210957	14.817165
H	7.215716	6.939716	13.481793
C	3.286774	5.978194	12.414520
H	2.965913	5.157176	12.033717
H	2.730236	6.704414	12.120807
H	3.256926	5.920638	13.372505
C	3.095820	7.573677	7.778286
H	3.664613	7.112383	8.413527
H	2.823890	6.933791	7.100354

C	4.129811	8.185631	4.731862
H	3.729644	8.347295	3.874628
H	4.044591	7.255427	4.953855
H	5.060679	8.423472	4.701124
C	1.862974	8.093372	8.495494
H	1.344864	7.352764	8.818237
N	1.191461	0.905659	13.205156
C	2.096658	1.989910	13.650849
H	2.072412	2.050851	14.619080
H	1.787026	2.834627	13.288830
C	0.721492	1.027542	11.811724
H	0.554202	0.143043	11.451413
H	1.413881	1.442283	11.273819
C	3.528367	1.746144	13.200033
H	3.849228	0.925126	13.580836
H	4.084906	2.472364	13.493747
H	3.558215	1.688588	12.242049
S	1.344321	6.319382	1.568977
N	3.203622	4.429264	1.398896
C	2.223426	5.065764	0.771852
C	3.969394	3.341627	0.759898
H	3.400601	2.880333	0.124657
H	4.241324	2.701741	1.437830
C	3.633831	4.788988	2.757834
H	3.443146	5.726810	2.914936
H	4.591910	4.662026	2.832970
C	2.935403	3.953581	3.806323
H	3.335570	4.115245	4.663556
H	3.020623	3.023377	3.584330
H	2.004534	4.191422	3.837060
C	5.202240	3.861322	0.042691
H	5.731168	4.382711	0.650610
S	1.293253	8.349835	15.681230
S	1.550519	4.628255	16.211622
C	0.743778	8.418394	14.021407
H	4.682601	4.408950	16.377946
H	5.470277	3.120714	16.796317
C	13.836420	1.852791	11.719512
H	13.122273	1.396577	12.172036
H	13.602580	1.978907	10.797388
H	13.979926	2.707666	12.132760
Pb	13.873713	6.425691	16.400316
S	14.005765	7.224871	13.491015
C	11.034608	0.890423	9.298083
H	10.465815	1.351717	8.662842
H	11.306538	1.530309	9.976015
C	10.000617	0.278469	12.344507
H	10.400783	0.116805	13.201741
H	10.085836	1.208673	12.122514
H	9.069748	0.040628	12.375245

C	12.267453	0.370728	8.580875
H	12.785564	1.111336	8.258132
Pb	6.808500	6.270459	7.862131
S	8.608540	4.346315	7.143045
S	8.409535	6.376768	10.107161
S	8.865805	8.067895	7.673437
S	6.940551	5.471279	4.952830
N	10.268836	8.266886	9.937081
C	9.288640	7.630386	9.310036
N	8.506747	3.326391	4.666972
C	8.059065	4.277756	5.483222
C	10.699045	7.907162	11.296018
H	10.508360	6.969340	11.453121
H	11.657124	8.034124	11.371154
C	9.411944	2.242140	5.112665
H	9.387699	2.181199	6.080895
H	9.102313	1.397423	4.750646
H	12.796382	8.313439	9.188794
H	11.997887	8.287200	7.839761
C	8.036778	3.204508	3.273540
H	7.869489	4.089007	2.913229
H	8.729167	2.789767	2.735634
C	6.771207	2.379259	3.181328
H	6.057059	2.835474	3.633851
H	6.537366	2.253143	2.259204
H	6.914712	1.524384	3.594576
C	10.843654	2.485906	4.661849
H	11.164514	3.306924	5.042652
H	11.400192	1.759686	4.955562
H	10.873501	2.543462	3.703864
Pb	0.256714	2.038409	0.676053
S	0.124663	1.239229	3.585354
C	0.294007	6.611309	5.356857
H	1.008155	7.067524	4.904333
H	0.527848	6.485193	6.278981
H	0.150502	5.756434	4.943609
S	12.837174	0.114265	1.395139
S	12.579909	3.835845	0.864747
C	13.386649	0.045706	3.054962
H	9.447827	4.055150	0.698423
H	8.660150	5.343386	0.280052
S	12.786106	2.144718	15.507392
N	10.926805	4.034836	15.677473
C	11.907001	3.398336	16.304517
C	10.161033	5.122473	16.316471
H	10.729827	5.583767	16.951712
H	9.889103	5.762359	15.638539
C	10.496596	3.675112	14.318535
H	10.687282	2.737290	14.161433
H	9.538517	3.802074	14.243399

C	11.195025	4.510519	13.270046
H	10.794858	4.348855	12.412813
H	11.109805	5.440723	13.492039
H	12.125893	4.272678	13.239309
C	8.928188	4.602778	17.033678
H	8.399260	4.081389	16.425759
N	12.938966	7.558441	3.871213
C	12.033770	6.474190	3.425520
H	12.058015	6.413249	2.457290
H	12.343401	5.629473	3.787539
C	13.408936	7.436558	5.264645
H	13.576225	8.321057	5.624956
H	12.716546	7.021817	5.802550
C	10.602060	6.717956	3.876336
H	10.281199	7.538974	3.495533
H	10.045522	5.991736	3.582622
H	10.572213	6.775512	4.834320
I	9.669140	0.374029	0.443303
I	7.214162	1.376432	14.943018
C	7.432153	0.326714	16.770702
I	6.916266	7.087668	2.133351
C	6.698275	8.137386	0.305667
I	4.461288	8.090071	16.633066
I	0.148948	2.855618	6.404834
I	2.353854	3.858021	8.981487
C	0.366939	3.905336	8.232518
I	13.981479	5.608482	10.671535
I	11.776574	4.606079	8.094882
C	13.763489	4.558764	8.843852
	PBETCA02		
H	7.485869	0.389580	3.889912
Pb	5.099643	5.773751	6.180745
S	7.619179	6.485435	5.406823
S	5.441960	8.517531	5.534952
N	8.027212	9.104641	5.239749
C	7.108921	8.154561	5.381745
C	9.453586	8.813657	5.039626
H	9.852831	9.521121	4.473251
H	9.544433	7.953186	4.557967
C	10.220773	8.733023	6.349148
H	11.181017	8.671051	6.162343
H	9.933153	7.940775	6.848352
H	10.043389	9.536968	6.881393
C	7.687082	10.539694	5.240579
H	8.357115	11.033525	5.776495
H	6.802845	10.665144	5.667914
C	7.648699	11.109975	3.843867
H	6.930224	10.681236	3.335071
H	8.507293	10.945155	3.400348
S	5.831083	3.237868	4.966884

S	4.076844	5.266224	3.671395
N	4.110248	2.681960	3.023443
C	4.619716	3.625028	3.815633
C	4.615292	1.293651	3.054168
H	5.594231	1.306483	3.201470
H	4.445193	0.865566	2.177577
C	3.950092	0.467444	4.156093
H	2.993347	0.378957	3.965161
H	4.071787	0.916003	5.018559
C	3.057540	2.955415	2.013691
H	2.549117	3.760891	2.282089
H	2.428527	2.192499	1.982726
C	3.650214	3.173945	0.629434
H	4.239324	2.424632	0.404291
H	4.164281	4.008250	0.622459
H	-1.155285	3.230774	8.274618
H	4.360557	11.264115	4.189416
H	6.401495	0.421985	0.037475
Pb	7.140581	5.912349	2.028804
S	9.660118	5.200665	1.254883
S	7.482899	3.168569	1.383011
N	10.068150	2.581459	1.087808
C	9.149860	3.531539	1.229805
C	11.494524	2.872443	0.887685
H	11.893769	2.164979	0.321310
H	11.585371	3.732914	0.406027
C	12.261711	2.953077	2.197207
H	13.221955	3.015049	2.010403
H	11.974092	3.745325	2.696411
H	12.084327	2.149132	2.729453
C	9.728020	1.146406	1.088639
H	10.398053	0.652575	1.624555
H	8.843783	1.020956	1.515973
S	7.872021	8.448232	0.814943
C	5.991030	11.218656	0.004152
H	6.112725	10.770097	0.866618
C	5.607760	0.576125	7.995807
H	4.889286	1.004864	7.487012
H	6.466355	0.740945	7.552289
S	2.035906	6.419876	7.823336
N	2.069309	9.004140	7.175384
C	2.578778	8.061072	7.967574
C	2.574354	10.392449	7.206108
H	3.553293	10.379617	7.353411
H	2.404255	10.820534	6.329517
H	0.952408	11.307143	8.117102
C	1.016602	8.730685	6.165632
H	0.508179	7.925209	6.434030
H	0.387589	9.493601	6.134667
C	1.609276	8.512155	4.781375

H	0.885653	8.455326	4.122678
H	2.198386	9.261468	4.556232
H	2.123343	7.677850	4.774400
H	5.444931	11.296520	8.041852
S	-0.508121	0.642385	5.406823
S	-2.685340	2.674481	5.534952
N	-0.100088	3.261591	5.239749
C	-1.018379	2.311511	5.381745
C	1.326286	2.970607	5.039626
H	1.725531	3.678071	4.473251
H	1.417133	2.110136	4.557967
C	2.093473	2.889973	6.349148
H	3.053717	2.828001	6.162343
H	1.805853	2.097725	6.848352
H	1.916089	3.693918	6.881393
C	-0.440218	4.696644	5.240579
H	0.229815	5.190475	5.776495
H	-1.324455	4.822094	5.667914
C	-0.478601	5.266925	3.843867
H	-1.197076	4.838186	3.335071
H	0.379993	5.102105	3.400348
H	-0.641431	6.232630	3.889912
Pb	-3.027657	11.616801	6.180745
S	-2.296217	9.080918	4.966884
S	12.204144	11.109274	3.671395
N	12.237548	8.525010	3.023443
C	12.747016	9.468078	3.815633
C	12.742592	7.136701	3.054168
H	13.721531	7.149533	3.201470
H	12.572493	6.708616	2.177577
C	12.077392	6.310494	4.156093
H	11.120647	6.222007	3.965161
H	12.199087	6.759053	5.018559
H	12.487857	5.421065	4.189416
C	11.184840	8.798465	2.013691
H	10.676417	9.603941	2.282089
H	10.555827	8.035549	1.982726
C	11.777514	9.016995	0.629434
H	12.366624	8.267682	0.404291
H	12.291581	9.851300	0.622459
H	6.972015	9.073824	8.274618
Pb	-0.986719	0.069299	2.028804
S	-0.255279	2.605182	0.814943
S	1.532818	11.043715	1.254883
S	-0.644401	9.011619	1.383011
N	1.940850	8.424509	1.087808
C	1.022560	9.374589	1.229805
C	3.367224	8.715493	0.887685
H	3.766469	8.008029	0.321310
H	3.458071	9.575964	0.406027

C	4.134411	8.796127	2.197207
H	5.094655	8.858099	2.010403
H	3.846792	9.588375	2.696411
H	3.957027	7.992182	2.729453
C	1.600720	6.989456	1.088639
H	2.270753	6.495625	1.624555
H	0.716483	6.864006	1.515973
C	-2.519540	6.419175	7.995807
H	-3.238014	6.847914	7.487012
H	-1.660945	6.583995	7.552289
H	-2.682369	5.453470	8.041852
C	14.118330	5.375606	0.004152
H	14.240025	4.927047	0.866618
H	14.528795	6.265035	0.037475
S	10.163206	0.576826	7.823336
N	10.196609	3.161090	7.175384
C	10.706078	2.218022	7.967574
C	10.701654	4.549399	7.206108
H	11.680593	4.536567	7.353411
H	10.531555	4.977484	6.329517
H	9.079708	5.464093	8.117102
C	9.143902	2.887635	6.165632
H	8.635479	2.082159	6.434030
H	8.514889	3.650551	6.134667
C	9.736576	2.669105	4.781375
H	9.012953	2.612276	4.122678
H	10.325686	3.418418	4.556232
H	10.250643	1.834800	4.774400
	IPTCPB01		
C	-1.946032	6.791301	18.327690
H	-1.711811	7.510153	18.953017
H	-1.127387	6.356295	18.009833
H	-1.202124	7.620164	15.897017
C	7.359163	0.557614	19.692606
H	6.424799	0.303161	19.842186
H	7.530324	1.430985	20.101873
C	7.619973	0.634874	18.244590
H	8.600954	0.823824	18.300682
C	7.250887	1.799649	17.473838
H	7.682278	2.593241	17.851943
H	6.278105	1.913859	17.498768
H	7.540534	1.682079	16.545196
H	9.854968	0.577769	16.360299
H	5.670387	1.247913	0.367717
H	5.585049	2.846014	0.403035
C	2.579506	3.907496	0.367717
H	2.668795	3.013970	0.762442
H	1.717037	4.294635	0.627404
H	3.305573	4.480226	0.691807
Pb	4.019979	5.563543	16.015227

S	1.672947	5.536754	17.659359
S	6.855385	5.535830	16.330383
S	3.877470	3.580654	17.841348
S	5.057280	7.451956	17.647101
C	6.661457	7.001246	17.189221
C	2.284769	4.064535	18.302760
N	1.563642	3.336446	19.156612
N	7.713041	7.757888	17.527853
C	1.969715	2.012113	19.711304
H	1.223748	1.750102	20.324166
C	3.204948	2.059141	20.594241
H	4.006059	2.113726	20.033316
C	2.003228	0.925438	18.647625
H	1.239030	1.034609	18.045150
H	1.956568	0.046188	19.077667
H	2.836184	0.995139	18.136560
H	7.948898	8.284430	20.095641
C	9.103516	7.347235	17.201686
H	8.893725	6.491499	16.730094
H	9.260093	6.132913	18.784740
C	9.709621	8.039214	16.113077
H	9.119532	7.996385	15.329860
C	0.228235	3.802524	19.626126
H	0.100933	4.726282	19.264642
C	-0.875917	2.940070	19.031962
H	-0.783143	2.917396	18.055538
H	-1.748666	3.318811	19.268797
H	-0.806739	2.029748	19.387214
Pb	4.113279	1.364643	15.147248
S	6.460311	1.337854	13.503116
S	1.277873	1.336930	14.832091
S	3.075979	3.253056	13.515373
C	1.471802	2.802346	13.973253
N	0.420217	3.558988	13.634621
C	0.774095	4.756514	11.469868
H	1.708459	4.502061	11.320288
H	0.602934	5.629885	11.060601
H	0.184361	4.085530	11.066833
C	0.513286	4.833774	12.917884
H	-0.467696	5.022724	12.861792
C	0.882371	5.998549	13.688636
H	0.450980	6.792141	13.310532
H	1.855153	6.112759	13.663706
H	0.592725	5.880979	14.617278
C	-0.970258	3.148335	13.960788
H	-0.760467	2.292599	14.432381
H	-1.126835	1.934013	12.377735
C	-1.576363	3.840314	15.049398
H	-0.986274	3.797485	15.832614
H	-1.721710	4.776669	14.802175

H	8.032326	0.527382	11.897833
H	8.840916	0.095735	9.760087
H	7.252380	0.281326	9.695685
S	4.255788	7.779554	13.321127
C	5.848490	8.263435	12.859714
N	6.569616	7.535346	12.005863
C	6.163544	6.211013	11.451171
H	6.909510	5.949002	10.838309
C	4.928310	6.258041	10.568234
H	4.127200	6.312626	11.129158
H	4.887566	5.446813	10.019774
H	4.972903	7.044914	9.984457
C	6.130031	5.124338	12.514850
H	6.894229	5.233509	13.117324
H	6.176691	4.245088	12.084808
H	5.297074	5.194039	13.025914
C	7.905023	8.001424	11.536348
C	7.978447	8.106396	10.019774
H	7.889158	7.212870	9.625050
C	9.009176	7.138970	12.130512
H	8.916402	7.116296	13.106937
H	9.881925	7.517711	11.893678
H	8.939997	6.228648	11.775260
C	10.079291	2.592401	12.834784
H	9.845069	3.311253	12.209457
H	9.260645	2.157395	13.152642
H	9.335383	3.421264	15.265457
Pb	5.325626	2.834258	4.759756
S	7.672659	2.861046	3.115624
S	2.490220	2.861970	4.444600
S	5.468135	4.817146	2.933635
S	4.288326	0.945844	3.127881
C	2.684149	1.396554	3.585762
C	7.060837	4.333265	2.472223
N	7.781963	5.061354	1.618371
N	1.632565	0.639912	3.247130
C	7.375891	6.385687	1.063679
H	8.121857	6.647698	0.450817
C	6.140657	6.338659	0.180742
H	5.339547	6.284074	0.741667
C	7.342378	7.472362	2.127358
H	8.106576	7.363191	2.729833
H	7.389038	8.351612	1.697316
H	6.509422	7.402661	2.638423
H	1.396708	0.113370	0.679342
C	0.242089	1.050565	3.573297
H	0.451881	1.906301	4.044889
H	0.085512	2.264887	1.990243
C	-0.364016	0.358586	4.661906
H	0.226074	0.401415	5.445123

C	9.117370	4.595276	1.148857
H	9.244673	3.671518	1.510341
C	10.221523	5.457730	1.743021
H	10.128749	5.480404	2.719445
H	11.094272	5.078989	1.506186
H	10.152344	6.368052	1.387769
H	3.675219	7.149887	20.407266
H	3.760556	5.551786	20.371948
C	6.766100	4.490304	20.407266
H	6.676811	5.383830	20.012541
H	7.628569	4.103165	20.147578
H	6.040033	3.917574	20.083176
C	1.986442	7.840186	1.082377
H	2.920806	8.094639	0.932797
H	1.815281	6.966815	0.673109
C	1.725633	7.762926	2.530393
H	0.744651	7.573976	2.474300
C	2.094718	6.598151	3.301145
H	1.663328	5.804559	2.923040
H	3.067501	6.483941	3.276215
H	1.805072	6.715721	4.229787
H	-0.509362	7.820031	4.414684
C	11.291638	1.606499	2.447293
H	11.057417	0.887647	1.821966
H	10.472992	2.041505	2.765150
H	10.547730	0.777636	4.877966
C	-0.733685	5.805399	7.940198
H	-0.499464	5.086547	8.565525
H	0.084960	6.240405	7.622341
H	0.010223	4.976536	5.509525
S	5.089818	0.618246	7.453856
C	3.497116	0.134365	7.915268
N	2.775989	0.862454	8.769120
C	3.182062	2.186787	9.323812
H	2.436095	2.448798	9.936674
C	4.417296	2.139759	10.206749
H	5.218406	2.085174	9.645825
H	4.458039	2.950987	10.755209
H	4.372702	1.352886	10.790526
C	3.215575	3.273462	8.260133
H	2.451377	3.164291	7.657659
H	3.168915	4.152712	8.690175
H	4.048531	3.203761	7.749069
C	1.440583	0.396376	9.238635
C	1.367159	0.291404	10.755209
H	1.456448	1.184930	11.149933
C	0.336430	1.258830	8.644470
H	0.429204	1.281504	7.668046
H	-0.536319	0.880089	8.881305
H	0.405609	2.169152	8.999723

Pb	5.232326	7.033158	5.627735
S	2.885294	7.059946	7.271867
S	8.067732	7.060870	5.942892
S	6.269627	5.144744	7.259610
C	7.873804	5.595454	6.801729
N	8.925388	4.838812	7.140362
C	8.571511	3.641286	9.305115
H	7.637146	3.895739	9.454695
H	8.742672	2.767915	9.714382
H	9.161245	4.312270	9.708149
C	8.832320	3.564026	7.857099
H	9.813302	3.375076	7.913191
C	8.463235	2.399251	7.086347
H	8.894625	1.605659	7.464451
H	7.490452	2.285041	7.111277
H	8.752881	2.516821	6.157705
C	10.315863	5.249465	6.814194
H	10.106072	6.105201	6.342602
H	10.472440	6.463787	8.397248
C	10.921969	4.557486	5.725585
H	10.331879	4.600315	4.942368
H	11.067315	3.621131	5.972808
H	1.313280	7.870418	8.877150
H	0.504690	8.302065	11.014896
H	2.093226	8.116474	11.079298
